# Biomechanical Regulatory Factors and Therapeutic Targets in Keloid Fibrosis

**DOI:** 10.3389/fphar.2022.906212

**Published:** 2022-05-09

**Authors:** Fan Feng, Mingying Liu, Lianhong Pan, Jiaqin Wu, Chunli Wang, Li Yang, Wanqian Liu, Wei Xu, Mingxing Lei

**Affiliations:** ^1^ National Innovation and Attracting Talents “111” Base, Key Laboratory of Biorheological Science and Technology, Ministry of Education, College of Bioengineering, Chongqing University, Chongqing, China; ^2^ School of Comprehensive Health Management, Xihua University, Chengdu, China; ^3^ Chongqing Clinical Research Center for Dermatology, Chongqing Key Laboratory of Integrative Dermatology Research, Department of Dermatology, Chongqing Hospital of Traditional Chinese Medicine, Chongqing, China

**Keywords:** keloid fibrosis, fibroblast, mechanotransduction, biomechanical factor, matrix force, targeted therapy

## Abstract

Keloids are fibroproliferative skin disorder caused by abnormal healing of injured or irritated skin and are characterized by excessive extracellular matrix (ECM) synthesis and deposition, which results in excessive collagen disorders and calcinosis, increasing the remodeling and stiffness of keloid matrix. The pathogenesis of keloid is very complex, and may include changes in cell function, genetics, inflammation, and other factors. In this review, we aim to discuss the role of biomechanical factors in keloid formation. Mechanical stimulation can lead to excessive proliferation of wound fibroblasts, deposition of ECM, secretion of more pro-fibrosis factors, and continuous increase of keloid matrix stiffness. Matrix mechanics resulting from increased matrix stiffness further activates the fibrotic phenotype of keloid fibroblasts, thus forming a loop that continuously invades the surrounding normal tissue. In this process, mechanical force is one of the initial factors of keloid formation, and matrix mechanics leads to further keloid development. Next, we summarized the mechanotransduction pathways involved in the formation of keloids, such as TGF-β/Smad signaling pathway, integrin signaling pathway, YAP/TAZ signaling pathway, and calcium ion pathway. Finally, some potential biomechanics-based therapeutic concepts and strategies are described in detail. Taken together, these findings underscore the importance of biomechanical factors in the formation and progression of keloids and highlight their regulatory value. These findings may help facilitate the development of pharmacological interventions that can ultimately prevent and reduce keloid formation and progression.

## Introduction

Skin is the largest organ of the human body, covering the entire surface of the human body, with the functions of protection, excretion, body temperature regulation, and others (Hsu et al., 2018a). As the interface between the external environment and internal tissue, skin is easily damaged by infection, disease, trauma, and other factors. When the skin is injured, the healing process can be categorized into hemostasis, inflammation, proliferation, re-epithelialization, and remodeling (Rodrigues et al., 2019). It should be noted that these five processes are only artificially divided. In fact, the healing process after injury is continuous and indivisible. Typically, after the completion of these five processes, the dermal tissue ends up filled with extracellular matrix (ECM) components, but does not overgrow, instead forming a filler tissue that differs from the normal skin structure, which is known as a physiological scar. When the skin healing process is maladjusted, the abovementioned process is further disordered, forming scar hyperplasia, usually accompanied by itching, local discomfort or pain, and other chronic symptoms, which is called a pathological scar. Pathological scarring has been reported in 32–60% of patients after surgery (Mahdavian Delavary et al., 2012).

Pathological scar, mainly including hypertrophic scar and keloid, are skin fibroproliferative disease characterized by an abnormal wound healing process, massive production of ECM mainly composed of collagen disorders and calcinosis, and hyperplasia of dermal tissue (Lee and Jang, 2018). A hypertrophic scar refers to a scar that does not exceed the original injury site. It is generally red or pink, relatively hard and itchy, and often disappears spontaneously after several years. Different from a hypertrophic scar, a keloid has the characteristics of excessive growth beyond the scope of trauma, and is more likely to invade adjacent tissues and cannot be resolved by itself. In other words, a keloid has the characteristics of invasive tumor growth, and therefore, it tends to be considered as a “benign skin tumor” (Limandjaja et al., 2020). Keloids are more likely to relapse after treatment, and are mainly treated via simple surgery, with a high recurrence rate. Therefore, the treatment of keloid is more difficult and requires more attention.

Keloid formation and progression is a complex process, which is believed to be independently or jointly driven by changes in cellular function, genetics, inflammation, and other factors. Of particular interest is the recent evidence that biomechanical factors play a key role in the formation and progression of keloids. Here we begin with an introduction to the various factors involved in the progression of keloid pathology and then describe the recent advances in biomechanical regulation of keloids as accumulated evidence suggests that several mechanotransduction signaling pathways, such as TGF-β/Smad signaling pathway, Integrin signaling pathway, YAP/TAZ signaling pathway, and calcium ion pathway, are involved in the process of keloid fibrosis, and have been proved to play an important role in keloid development. Finally, some potential therapeutic concepts and strategies based on biomechanical factors are described.

## Keloid Pathogenesis Study

A keloid is a skin disorder caused by excessive proliferation of skin fibers. The exact mechanism underlying keloid formation is still not fully understood. Current studies suggest that changes in cell function, genetics, inflammation, and biomechanical factors all play an important role in keloid formation.

### Changes in Cellular Function

During the process of keloid formation, the function of many types of cell populations abnormally changes. Fibroblasts are the most abundant cell type in all connective tissues of the body. As far as skin is concerned, fibroblasts mainly form the ECM by constructing different proportions of collagen and elastin to maintain the structural integrity and normal physiological functions of the skin (Plikus et al., 2021; Xue et al., 2022). Fibroblasts, the primary effector cells in keloids, eventually lead to keloid formation by inducing a persistent inflammatory response and excessive ECM deposition (Suarez et al., 2015; Luo et al., 2017). This process is driven by many growth factors, including transforming growth factor -β (TGF-β), platelet-derived growth factor (PDGF), fibroblast growth factor β (FGF-β), and insulin-like growth factor I (IGF-I) (Andrews et al., 2016; Nagar et al., 2021). In keloids, the effects of these growth factors on fibroblasts contribute to the enhanced scar phenotype. Changes in fibroblast phenotypes are believed to be at the core of keloid formation. Studies have shown that the fibroblasts in the center of a keloid are usually in a resting state, whereas those in the periphery are in an abnormal proliferating state. Compared with normal skin fibroblasts, keloid fibroblasts have stronger anti-apoptosis and migration abilities (Wang et al., 2013; Jumper et al., 2017).

Some studies have suggested that keloid formation is associated with metabolic reprogramming of keloid fibroblasts, including the transition from oxidative phosphorylation to aerobic glycolysis. Compared with normal skin, the keloid tissue showed upregulated GLUT-1 expression and enhanced expressions of several glycolytic enzymes, such as HK1, HK2, PFK1, PFK2, PDK1, and PKM2, (Vinaik et al., 2020). Keloid fibroblasts also had higher basic glycolysis and glycolysis capacity and reduced basal oxidative respiration, maximum oxidative respiration, and reserve respiration capacity (Li et al., 2018; Li et al, 2020). GLUT-1-dependent glycolysis and ROS production mediate the proliferation of keloid fibroblasts, and GLUT-1 inhibitor WZB117 can reduce glycolysis and ROS production of keloid fibroblasts (Lu et al., 2021).

Although keloid fibroblasts are still believed to be responsible for keloid formation, recent studies have shifted focus to recognizing the potential role of abnormal epidermal cell populations in scar formation. Basic abnormalities found in keloid keratinocytes with regard to the secretion of wound healing mediators, differentially expressed genes, paracrine action of co-cultured cells, and epithelial-mesenchymal transition (EMT) all support a more active role of keratinocytes in keloid formation (Yan et al., 2015; Hahn et al., 2016; Li and Wu, 2016). In addition, co-culture of abnormal keloid keratinocytes and dermal fibroblasts induces a pro-fibrotic phenotype and increases the expression of pro-fibrotic factors (Lee et al., 2016).

### Genetic Factors

Recent studies have shown that race, genetic susceptibility, age, and gender are patient characteristics that may influence susceptibility to keloids. There are significant differences in scarring among different ethnicities, with worldwide keloid prevalence ranging from 0.09% in the United Kingdom to 16% in Congo, suggesting that individuals with darker skin are more likely to develop it (Kiprono et al., 2015). In addition, keloid has familial genetic characteristics, and family members of keloid patients have a higher disease incidence. For example, the prevalence rate of keloid in the first, second, and third degree relatives of Chinese keloid patients was 7.62, 0.38, and 0.035%, respectively (Lu et al., 2015). In addition, keloid can occur at all ages, but the incidence is highest between the ages of 10 and 30 years (Young et al., 2014).

There is increasing evidence indicating that various different and reversible epigenetic modifications, represented by DNA methylated histone modifications and non-coding RNAs, play key roles in the gene regulation of keloid and downstream fibroblast functions (He et al., 2017; Xu et al., 2019; Lv et al., 2020). Histone modification refers to the processes such as methylation, acetylation, phosphorylation, adenylation, ubiquitination, and ADP ribosylation by related enzymes (Wang et al., 2016a). DNA methylation is involved in keloid formation, and includes processes such as cell proliferation, invasion of myofibroblasts, activation of collagen deposition and disorder (Nyika et al., 2022). Russell et al. found that abnormal DNA methylation and histone acetylation modification of keloid fibroblasts can affect the stability of gene expression, and DNA methylation of HOXA9 and HOXA10 can alter the wound healing ability of keloid fibroblasts (Russell et al., 2010). Liu et al. (2018) further found that SFRP1 promoter methylation promotes keloid formation by promoting Wnt/β-catenin signaling pathway activity and β-catenin and α-SMA mRNA and protein expressions. Functional non-coding RNAs, such as microRNAs (miRNAs) and long non-coding RNAs (lncRNAs), play an important role in the regulation of keloid gene expression (Lv et al., 2020). Wang et al. (2019) found that miR-152-3p expression was significantly upregulated in keloid tissue and keloid fibroblasts compared with normal skin tissue. Further studies showed that miR-152-3p regulates cell proliferation, invasion, and ECM expression by targeting FOXF1 in keloid fibroblasts. Jin et al. (2019) showed that lncRNA HOXA11-AS induced collagen I synthesis *via* Smad5 signal transduction mediated by sponging miR-124-3p to promote keloid formation.

### Inflammatory Factors

Studies have shown that inflammatory mediators and inflammatory cells are involved in the entire process of wound healing (Ogawa, 2017; Rodrigues et al., 2019). It is believed that pathological scars are caused by excessive and persistent inflammation, and the intensity of inflammation is positively correlated with the final scar size (Huang et al., 2014). Various inflammatory cells, including (Zhou et al., 2021), mast cells (Bagabir et al., 2012), lymphocytes (Chen et al., 2018), and monocytes (Limandjaja et al., 2019), have been reported to be involved in keloid formation. Among these, macrophages are important cells that initiate an inflammatory response during wound healing and lead to excessive spreading of inflammation. In keloid tissues, macrophages upregulate M2-related genes highly associated with tissue repair and remodeling (Jin et al., 2018). These macrophages further promote the transformation of fibroblasts into myofibroblasts by secreting TGF-β and PDGF, which in turn promotes collagen deposition and scarring (Hesketh et al., 2017). TGF-β is a representative pro-fibrotic cytokine that is most closely related to keloid formation (Berman et al., 2017). In contrast, TGF-β-secreted factors can indirectly contribute to fibrosis through signaling pathways in an inflammatory microenvironment (Hahn et al., 2016). In addition, pro-inflammatory factors such as interleukin (IL)-1α, IL-1β, and IL-8, CKLF-1, and COX-1 are upregulated in the keloid tissue, suggesting that in patients with keloids, pro-inflammatory genes in the skin are sensitive to trauma, which in turn may promote chronic inflammation and lead to excessive keloid growth (Abdou et al., 2014; Lin et al., 2020; Wu et al., 2020).

## Biomechanical Factors in Keloid Formation

The abovementioned studies mainly focus on the role of biological factors in keloid formation and have made great progress. They initially revealed the mechanism of occurrence and development of keloids, but the clinical efficacy was not significant after intervention with different targets, indicating that previous studies were incomplete. Recent studies have focused on the role of the external environment, particularly the local mechanical wound environment. Current scientific basis and clinical evidence clearly indicate that biomechanical factors play an important role in keloid formation, contracture, and abnormal keloid development and treatment. Next, we will summarize the research in this area and explore the role of biomechanical factors in keloid formation.

### Biomechanical Factors Associated With Skin Wound Healing

Skin injury is a common phenomenon in the human body, and the recovery of anatomical continuity and functional reconstruction of tissue after injury is a complex and dynamic process. Early observations in anatomy and surgery suggest the importance of mechanical tension in wound healing outcomes. For example, Langer lines in the human skin correspond to tension bands that occur naturally in the skin because of the interaction of collagen fibrils and fibroblasts. Conventional assumptions point to the alignment of the underlying ECM, cell orientation and contraction, and muscle involvement in the formation of these invisible lines. Surgical incisions made parallel to these lines reduce tension and heal more easily, with less scarring than incisions made perpendicular to them (Wong et al., 2011; Yazdani Abyaneh et al., 2014). The mechanical properties of the skin are mainly associated with the composition and tissue of ECM in the dermis (Shook et al., 2018). Collagen, the most abundant protein in ECM, exists in the dermis as fibrin; it provides tensile strength and determines the stiffness and mechanical strength of skin tissues (Nyström and Bruckner-Tuderman, 2019). The skin is also affected by tension, which plays an important role in maintaining homeostasis. There are many sources of skin tension, such as stretching of fore-chest skin caused by breathing and stretching caused by joint movement. Normal mechanical forces on wounds and scars induce tension homeostasis, allowing cells and ECM in tissues to progress normally at different stages of wound healing. The balance of internal and external mechanical forces, represented by ECM, cytoskeleton, intracellular signaling, and applied tensile stress, ensures normal healing of the skin after injury, whereas disruption of this balance may lead to pathological scarring (Harn et al., 2019).

### Tension in Keloid Genesis and Development

Research has shown that keloids clearly tend to occur in areas of high stress. Rei Ogawa’s map of 1,500 keloid lesions in 483 Japanese patients showed 733 (48.9%) in the anterior thoracic region and 403 (26.9%) in the scapular region, which are the regions usually exposed to continuous or intermittent tension stimulation during daily life activities. Keloid incidence was lower in areas where the skin stretched and contracted less (such as the parietal area or the anterior leg), even in patients with multiple or large keloids (Ogawa et al., 2012). Dohi et al. (2019) also showed that changes in human postural positions (i.e., standing, sitting, and supine) are associated with dynamic changes in local stress/strain distributions, especially in areas prone to keloid formation. In terms of shape, keloids often grow in the direction of skin tension and take on a specific shape, namely, the typical butterfly, crab claw, and dumbbell shape appears on the shoulder, chest, and upper arm, respectively (Ogawa et al., 2003; Jumper et al., 2017). In the fore-chest, for example, keloids often grow horizontally because the pectoralis major muscles contract in a horizontal direction. In addition, elongated vaccine keloids, which are common in the upper arm, are associated with widespread vaccination during childhood: shoulder keloids tend to grow along the long axis because of the tension exerted on the vaccine wound by arm movement. Three-dimensional finite element models based on clinical samples showed that respiratory movements caused stress in the area around the chest keloid, and the stress was concentrated in the area around the bilateral ends of the keloid, resulting in the keloid possibly taking on a crab or butterfly shape (Nagasao et al., 2013). Keloid, as a result of malregulated wound healing, is usually accompanied by inflammation in the early stage, which to some extent promotes excessive proliferation of fibroblasts, increased production of fibroblast products, and high expression of cytokines related to inflammatory reactions in these inflammation-infiltrated areas. Studies have also shown that mechanical force stimulation can continuously activate acute inflammatory pathways, thereby promoting the prolongation of inflammatory period, leading to wound fibrosis, and promoting hyperplasia of fibrous tissue to form keloids (El Ayadi et al., 2020).

In addition, mechanical stimulation is also one of the important factors causing functional changes in keloid cells. Suarez et al. established a new three-dimensional collagen lattice model using photogrammetry and *in vitro* technology to simulate the tension experience of normal skin and keloid tissue *in vivo*. The results showed that three tension-related genes (Hsp27, PAI-2, and integrin α2β1) were up-regulated in keloid fibroblasts under stress, which promoted ECM synthesis and cell proliferation (Suarez et al., 2014). Kiya et al. focused on endothelial cells in keloid dermal microvessels. Dermal microvascular endothelial cells release endothelin-1 (ET-1) in response to continuous uniaxial tensile stress, contributing to fibroblast-to-myofibroblast differentiation, collagen synthesis, and contractile properties via the RhoA/Rho kinase pathway (Kiya et al., 2017). Stretch tension can promote a high expression of integrin αVβ3 in keloid-derived mesenchymal stem cells (MSCs), suggesting that stretch tension may affect MSC secretion (Song et al., 2019). These results suggest that mechanical stimulation can cause functional changes related to wound healing in cells, leading to the development of keloids. Lipid metabolism and their metabolites play an important role in keloid development and are believed to be involved in mechanical transduction (Huang and Ogawa, 2013). Caveolin-1 (CAV-1), as a biofilm marker protein and one of the main scaffold proteins of cell membranes, plays an important role in cell signal transduction, cell adhesion, intracellular cholesterol transport, and lipid metabolism, and is also related to cellular mechanical regulation (Hsu et al., 2018b; Vykoukal et al., 2020). CAV-1 shows low expression in keloid fibroblasts (Zhang et al., 2011). Low expression of CAV-1 decreases cell stiffness, but increases the contractility and migration ability of keloid fibroblasts. In keloid fibroblasts, reduced CAV-1 leads to upregulation of the transcription factor RUNX2, which is a potential regulator of increased ECM production in keloids and is associated with fibrosis (Hsu et al., 2018b).

### Matrix Mechanics in keloid Genesis and Development

The influence of matrix mechanics on keloid progression has also attracted the attention of researchers. Stretching and tension of skin may result in overexpression of tension-related proteins in keloids. Keloid formation is also closely related to excessive cell proliferation and ECM deposition (Canady et al., 2013; Xue and Jackson, 2015). Histopathological examination of mature keloid lesions revealed the deposition of connective tissue ECM, particularly collagen I and III with dense fibrous structures. Collagen V and VI were also found to be abundant, especially in the early stages of development. Therefore, up-regulation of collagen VI gene expression can be used as an early biomarker in the process of keloid fibrosis (Andrews et al., 2016). Collagen fibers in keloid tissue are clumped rather than netted, and its fibers are thicker, more parallel, with less cross-linking between fibers (Syed et al., 2011). Another prominent protein found in keloid ECM is periostin, a stromal cell protein that promotes keloid formation by activating the RhoA pathway to promote TGF-β1 secretion (Zhang et al., 2014; Maeda et al., 2019). These pathological features lead to a large amount of keloid collagen production, pathological cross-linking, and deposition and remodeling of ECM, resulting in matrix stiffness of keloid that is different from that of normal skin. Therefore, matrix stiffness, which is as an important feature and marker of keloid pathology, has received increasing attention in clinical diagnosis of keloids. Changes in ECM content during keloid formation allow tissue stiffness to be characterized by acoustic imaging to observe information about target tissue stiffness. Using shear-wave elastography, researchers have found that the Young’s modulus of keloid tissue was around 124.6 KPa, which is much higher than 17.7 KPa of normal skin (Huang et al., 2020; Hang et al., 2021). These advances allow for the noninvasive assessment of progressive tissue stiffness and provide a valuable tool for the clinical understanding of progressive tension and maladaptive wound repair.

Matrix stiffening is not only the pathological result of keloid fibrosis but also an important clue indicating the progression of keloid fibrosis. Although stromal stiffness, an important feature and marker of keloid pathology, has received increasing attention and recognition in the clinical diagnosis of keloid, matrix mechanics has also been shown to facilitate the progression of keloids. Macarak et al. have reported that the higher the stiffness of the basal surface (3, 10, 25 KPa, >1,000 KPa), the higher the expression of the pro-fibrotic gene α-SMA in keloid fibroblasts (Macarak et al., 2021). Kenny et al. (2018) found that increased substrate stiffness specifically stimulated keratinocyte proliferation without affecting adhesion, survival, or terminal differentiation, and was mediated by epidermal growth factor (EGF) signaling. Deng et al. (2021) found that keloid fibroblasts are stiffer than normal fibroblasts when cultured on hydrogels that mimic tissue stiffness. Matrix stiffness contributes to the transformation of normal keloid fibroblasts to a fibrotic phenotype, such as that with higher YAP nuclear expression. In 2010, Kurokawa et al. (2010) constructed three-dimensional images of keloids and hypertrophic scar vessels and found that the number of capillaries in keloids was significantly lower than that in hypertrophic scar, and that the lumen was flatter. This is caused by the pressure of a large number of fibroblasts and collagen in keloids. Excessive proliferation of fibroblasts and a large amount of collagen deposition will also hinder the diffusion of oxygen around, resulting in tissue hypoxia (Lei et al., 2019). Importantly, hypoxia is believed to be a key mechanism underlying keloid formation. Previous studies have shown that keloid fibroblasts proliferate, migrate, invade, and increase collagen synthesis under hypoxic conditions (Zhang et al., 2014; Kang et al., 2020). In addition, hypoxia promotes EMT in keloid fibroblasts and inhibits apoptosis (Kim et al., 2019).

Based on the importance of matrix mechanics in the current process of keloid fibrosis, studies have been conducted to investigate whether targeting keloid matrix mechanics has the potential to treat or even reverse fibrosis. Obviously, the most direct approach toward altering keloid matrix mechanics is to inhibit and slow the increase in keloid stiffness during fibrosis. From this viewpoint, it is possible to reduce the stiffness of keloid matrix by influencing or reducing the factors that promote the stiffening of keloid matrix. As one of the main components of fiber matrix, collagen plays a key role in regulating the mechanical properties of ECM. Therefore, it is possible to reduce the stiffness of ECM and slow down the process of keloid fibrosis by reducing collagen synthesis in ECM or excessive deposition of collagen degraded by collagenase. Transmembrane protein 88 (TMEM88) belongs to the TMEM family and is involved in the regulation of tumorigenesis and fibrogenesis. Zhao et al. (2017a) found that overexpression of TMEM88 inhibited TGF-β1 expression in keloid fibroblasts, thereby decreasing collagen I expression. Tissue inhibitors of matrix metalloproteinases (MMPs) and tissue inhibitors of metalloproteinases (TIMPs) are two important regulators of degradation and remodeling of ECM. Aoki et al. (2014) promoted the degradation of collagen I in keloids by targeting TIMP-1 with small interfering RNA. Lysyl oxidase (LOX) plays an important role in collagen cross-linking, and helps increase tissue stiffness and the resistance of collagen-rich substrates to degradation. Kim (2021) found that keloid dermal fibroblasts showed the highest degree of expression of skin fibrosis markers, such as LOX and four LOX-like family enzymes, at the cell-matrix interface. Epidermal growth factor-mediated LOX expression was significantly reduced, thereby reducing the fibrotic phenotype of keloid fibroblasts and promoting collagen degradation.

**FIGURE 1:**
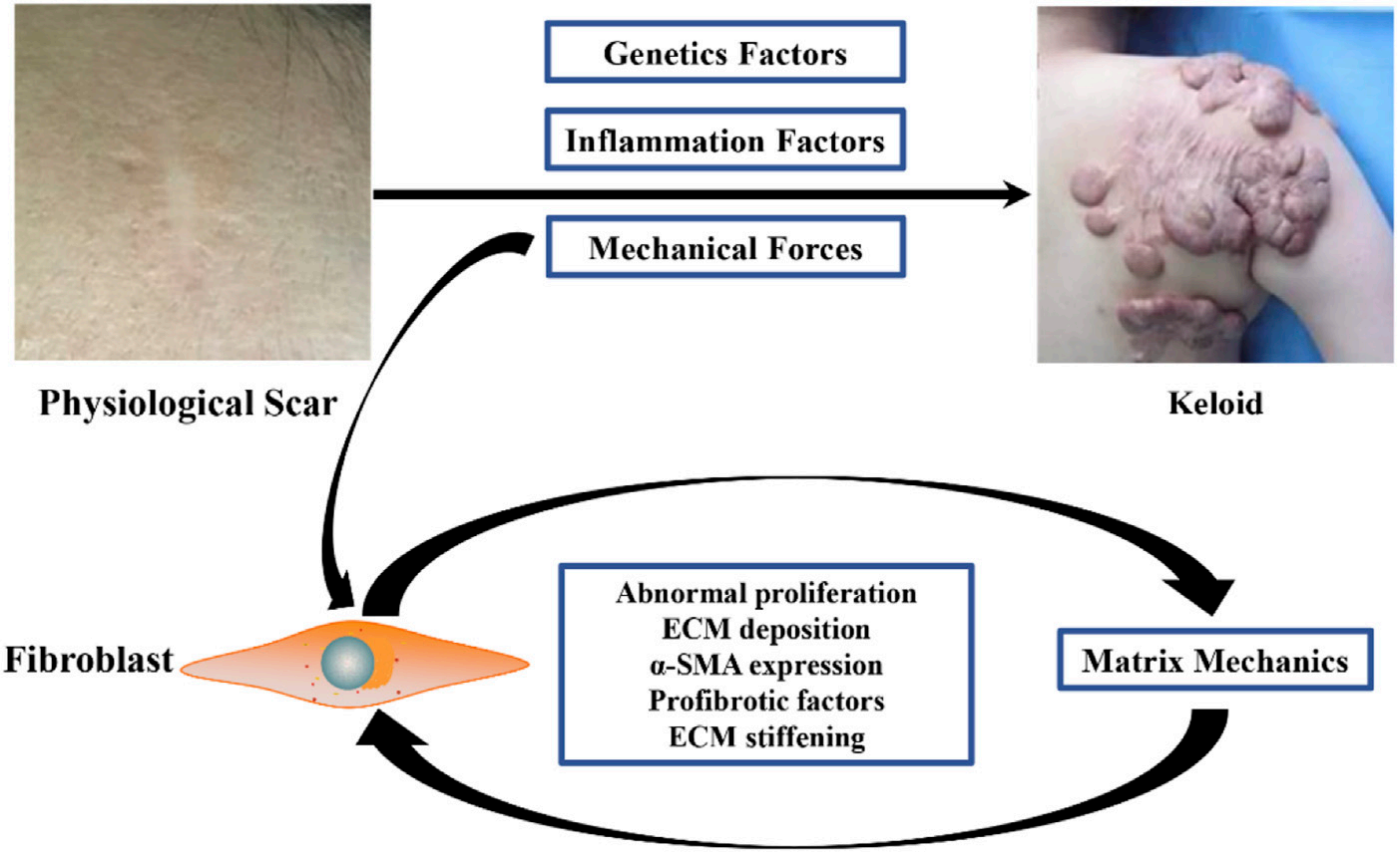
Causes of keloid matrix stiffening during fibrotic progression. Mechanical stimulation can lead to excessive proliferation of wound fibroblasts, deposition of ECM, secretion of more pro-fibrosis factors, and continuous increase of matrix stiffness of keloid ECM. Matrix mechanics resulting from elevated matrix stiffness further activates the fibrotic phenotype of keloid fibroblasts, thus forming a loop that continuously invades surrounding normal tissue.

In conclusion, current studies suggest that mechanical stimulation can lead to excessive proliferation of fibroblasts at the wound site, ECM deposition, and secretion of more pro-fibrotic factors, thus promoting the continuous increase of ECM stiffness in keloids. At the same time, the matrix mechanics of ECM further promotes the fibrotic phenotype of keloid fibroblasts, thus forming a loop and resulting in continuous invasion of the surrounding normal tissues by keloids. During this process, the mechanical force is one of the initiating factors of keloid formation, and matrix mechanics leads to further keloid development (Figure 1).

## Biomechanical Factors and Mechanotransduction Pathways in Keloids

**FIGURE 2:**
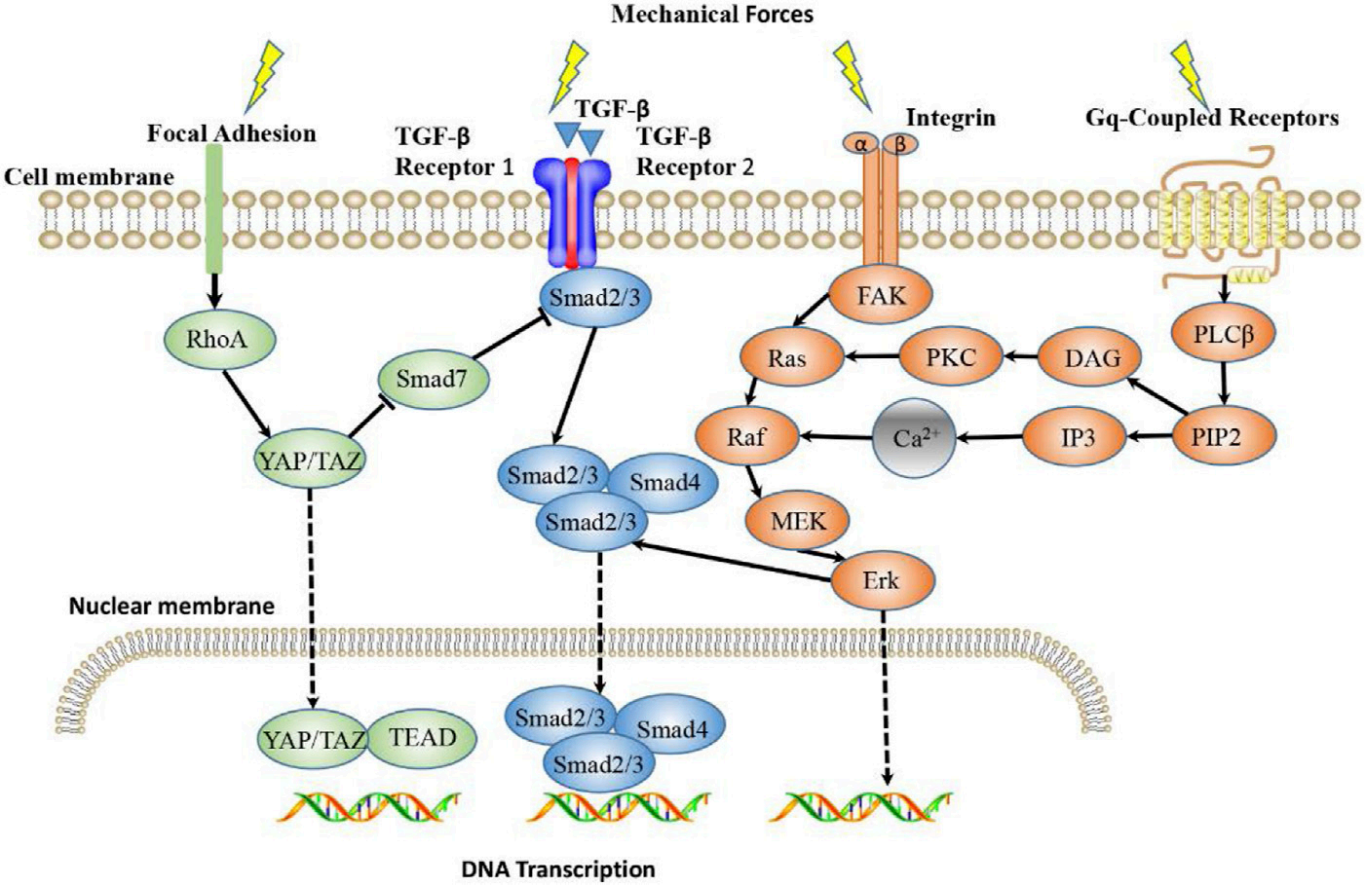
Main Mechanotransduction Signaling Pathways in keloid. TGF-β/Smad, integrin/FAK, YAP/TAZ signaling pathways and Calcium ion signaling are the main mechanotransduction signaling pathways that mediate mechanoresponsiveness. TGF-β senses mechanical forces and signals through Smads, where Smad2/3 binds to Smad4 and enters the nucleus, where they bind DNA and initiate trans-activation of target genes. Integrin signaling induces gene expression through FAK/Erk pathway, and also interferes with TGF-β/Smad signaling pathway. Mechanical stimulation can promote YAP to nuclear transfer and function. The Gq-coupled receptor activated by stretch stimulation can activate PLCβ and produce DAG and IP3. IP3 acts on the intracellular calcium pool to release Ca^2+^, resulting in the increase of intracellular free calcium ion concentration and the subsequent intracellular reaction of Raf/MEK/Erk pathway.

In the pathological process of gradual stiffening of keloid matrix, the cells in keloid sense changes in mechanical properties of the microenvironment and activate mechanical transduction pathways, thus transforming external mechanical stimuli into biochemical signals and ultimately guiding cellular behavior (Mierke, 2022). How matrix mechanics affects and regulates cell fate at a macro to micro scale is still unclear. However, some studies have found that some proteins involved in mechanical transduction pathways play a role in this process. Several mechanically sensitive pathways in keloid development and their effects on keloid progression will be introduced next (Figure 2).

### TGF-β/Smad Signaling Pathway in Keloids

As a classical signaling pathway, the TGF-β signaling pathway has been proved to be an important signaling pathway involved in fibrosis progression during mechanical regulation (Chen et al., 2019). The transforming growth factor superfamily includes TGF-β, bone morphogenetic protein (BMP), and growth differentiation factor (GDF), among which there are three different subtypes of TGF-β in the mammalian genome, namely TGF-β1, TGF-β2, and TGF-β3. Most of the biological functions of TGF-β superfamily are realized by binding to TGF-β receptor and activating the Smad signaling pathway (Frangogiannis, 2020). TGF-β1 is a stimulant of wound repair and tissue regeneration and a mediator of ECM production. During active wound healing, TGF-β is involved in various processes, including inflammation, angiogenesis, cell proliferation, collagen and matrix production, and wound remodeling (Pakyari et al., 2013). TGF-β is overexpressed in keloid the tissue and regulates the transformation of fibroblasts to myofibroblasts, and TGF-β1 has the highest proportion and the strongest activity. TGF-β1 can stimulate the proliferation and differentiation of keloid fibroblasts, up-regulate the expression of α-SMA and collagen, and promote the synthesis of ECM, thus leading to increased keloid tissue stiffness (Wang et al., 2016b; Zhang et al., 2020). TGF-β level is closely associated with the response of keloids to mechanical forces. Wang et al. (2006) found that keloid-derived fibroblasts exposed to equibiaxial strain produce higher levels of TGF-β1, TGF-β2, and collagen 1α at mRNA and protein levels than those produced by normal skin fibroblasts. Hypoxia induces the transformation of fibroblasts into myofibroblasts by activating TGF-β/Smad3 signaling pathway and increases collagen synthesis in keloid fibroblasts in a hypertrophic keloid microenvironment induced by high matrix stiffness (Zhao et al., 2017b).

### Integrin Signaling Pathway in Keloids

Integrin family is a kind of cell adhesion, signal pathway hub and the function of mechanical force signal sensing dimers proteins across the membrane. They are the link of ECM to the actin cytoskeleton in the cell and regulate specific signal transduction cascade reactions. Therefore, the protein is mediated the effect of matrix–cell and the cell–cell interaction, so as to promote the reshaping of the ECM (Bachman et al., 2015). Wound tension is an important factor in activating the integrin signaling pathway, which can directly activate integrin, convert mechanical signals into chemical signals in the cytoplasm, and promote the proliferation and differentiation of fibroblasts and secretion of collagen (Gauthier and Roca-Cusachs, 2018). Keloid fibroblasts showed up-regulated expressions of α1β1 and α2β1 integrins. Integrin α1β1 promoted fibroblast proliferation but inhibited collagen synthesis, whereas α2β1 had the opposite effect (Suarez et al., 2013). Song et al. analyzed integrin expression in keloid-derived mesenchymal stem cells, including α2, α3, α5, αV, α8, α10, α11, β1, and β3, and found that mechanical tension up-regulated the expression of integrin αV and integrin β3, thus up-regulating cell proliferation and collagen synthesis. Moreover, integrin αvβ3 is more sensitive to mechanical tension, and may be a new target for keloid treatment and prevention of keloid recurrence (Song et al., 2019). In addition, integrin also mediated the activation of TGF-β. In the process of TGF-β activation, integrin binds to the pre-domain and releases TGF-β using the stretching force generated by the actin cytoskeleton. At the same time, integrin can be abnormally expressed under the stimulation of TGF-β, promoting physiological and pathological changes in the body (Dong et al., 2017). During keloid formation, increased mechanical forces transmit high pressure through fibroblasts. This pressure is applied on integrins, causing latency-related peptides to unfold and release an active form of TGF-β (Leask, 2013).

### YAP/TAZ Signaling Pathway in Keloids

Yes-associated protein (YAP) and transcriptional coactivator with a PDZ-binding domain (TAZ) are among the activated mechanosensory pathways. YAP/TAZ can read a wide range of mechanical cues, such as ECM stiffness and topology, shear stress, and cell shape, and translate these into cell-specific biological effects (Zanconato et al., 2019). This ability to respond to different mechanical signals highlights YAP/TAZ’s central role as a general purpose mechanical sensor and effector. Mechanical transduction of YAP/TAZ is mainly controlled by its subcellular localization because YAP/TAZ activation requires its accumulation in the nucleus (Panciera et al., 2017). Gao et al. (2022) study showed that increased nuclear YAP/TAZ staining was observed in keloid tissue fibroblasts compared with that in normal skin. Activation of YAP-TAZ in the nucleus leads to the expression of pro-fibrosis genes, increased α-SMA expression, and excessive matrix deposition (Panciera et al., 2017). YAP/TAZ can be activated by matrix stiffness to further promote the transformation of fibroblasts into myofibroblasts and increase collagen deposition through a positive feedback loop, thereby enhancing the stiffness of the matrix microenvironment and leading to further YAP activation (Liu et al., 2015). Rho/Rho kinase signaling is a key upstream regulator of mechanical and receptor-mediated YAP and TAZ activation and also provides mechanosensory functional connectivity through integrin-based adhesion and tractor-producing actomyosin cytoskeleton. ROCK is a major downstream effector of Rho, driving cell contractility and mediating fibrosis pathology (Johan and Samuel, 2019). Inhibiting ROCK with drugs or inhibitors can alleviate the keloid fibrosis process caused by increased matrix stiffness, thereby disrupting or blocking the cellular response to tissue stiffness (Mun et al., 2014; Kiya et al., 2017).

### Calcium Ion Signaling in Keloids

It is believed that many signaling pathways are associated with Ca^2+^ influx, which is associated with a response to mechanical forces. The Gq-coupled receptor activated by stretch stimulation can activate PLCβ and produce DAG and IP3 (Jemal et al., 2014). IP3 acts on the intracellular calcium pool to release Ca^2+^, resulting in an increase in intracellular free calcium ion concentration and the subsequent intracellular reaction of the Raf/MEK/Erk pathway (Jain et al., 2018). In fibroblasts, uniaxial stretching increases intracellular Ca^2+^ levels (Sakamoto et al., 2010). The Piezo1 channel has been identified as a new mechanically activated cation channel (MAC) reportedly capable of modulating force-mediated cellular biological behavior. In hypertrophic scars, cycling mechanical stretching increased Piezo1 expression and promoted proliferation, migration, and differentiation of human skin fibroblasts and activated Piezo1-mediated influx of calcium (He et al., 2021). Yan et al. (2021) demonstrated phosphoproteome and biological evidence of abnormal calcium homeostasis and induction of abnormal platelet aggregation in keloid fibroblasts. These results suggest that the calcium pathway plays a role in mechanical transduction of keloids. There are also some studies showing that calcium antagonists are effective in treating keloids. Calcium antagonists reduce ECM production, induce collagenase synthesis, and inhibit IL-6, vascular endothelial growth factor, and fibroblast proliferation (Verhiel et al., 2015). As a calcium channel blocker, verapamil can increase the proteolytic activity of collagenase and regulate collagen metabolism in the ECM. Verapamil treatment increases procollagenase synthesis and decreases fibrous tissue production in keloids (Li and Jin, 2016; Srivastava et al., 2019).

## Clinical Control of Skin Tension for Keloid Prevention and Treatment

Biomechanical factors play an important role not only in the occurrence and development of keloids but also in its prevention and treatment. Many targeted biomechanical approaches have been developed for keloid prevention and treatment.

### Pharmacological Strategies

Several drugs have been developed to target mechanical transduction signaling pathways. TGF-β has been shown to play an important role in the fibrosis process of keloids, and therefore, multiple therapeutic strategies (including drugs, siRNA, shRNA, and miRNA, among others) have been designed to target TGF-β/Smad signaling to interfere with fibroblast-mediated keloid progression (Zhang et al., 2020; Marty et al., 2021). Ginsenoside Rg3 is a substance extracted from ginseng, a traditional Chinese medicine. Rg3 can inhibit the proliferation of keloid fibroblasts and inhibit the migration of keloid fibroblasts by up-regulating the expression of the anti-fibrosis gene TGF-β3 and down-regulating the expression of the pro-fibrosis gene α-SMA and connective tissue growth factor (Tang et al., 2018). Treatment with integrin αVβ3 inhibitor Cyclo (-RGDfK) resulted in a dramatic decrease of keloid-derived mesenchymal stem cell proliferation, collagen I, and collagen III for 48 h after either static culture or tensile culture (Song et al., 2019). Gao et al. have significantly inhibited the proliferation of keloid fibroblasts, reduced cell migration, induced apoptosis, and down-regulated collagen I production by targeting low endogenous YAP or TAZ knockdown. YAP/TAZ inhibitor verteporfin has shown similar but stronger inhibitory effect on fibroblasts (Gao et al., 2022).

### Suture of Intradermal Tension Reduction and Extradermal Tension Reduction Therapy

Surgeons have long known that optimizing incision design is an effective means to reduce wound mechanical tension and prevent postoperative scar hyperplasia. Surgical incisions are usually designed to be parallel to skin Langer’s lines, and vertical incision should not be made as far as possible to reduce skin tension. During plastic surgery, Z-plasty, W-plasty, and V-Y plasty are often used to repair scars, so as to disperse the mechanical tension of the incision and reduce the possibility of postoperative scar widening and recurrence (Ogawa, 2019). After keloid excision, degradable subcutaneous or fascial tensioning sutures can be used. Tension is placed on the deep and superficial fascia layers so that the wound edges can join naturally with very little tension, avoiding dermal sutures (Wang et al., 2014; Tsuge et al., 2020). Intralesional injection of Botulinum toxin type A (BoNT-A) is an effective clinical method for preventing and treating keloids. When administered locally, BoNT-A causes temporary paralysis of the wound muscle, thereby reducing disposal tension (Kasyanju Carrero et al., 2019). Skin tensioners can also be used to reduce the postoperative incision tensioning. The skin tensioning device is usually composed of adhesive tape fixing parts on both sides and an adjustable locking part in the middle. The fixing part is worn on both sides of the incision. By adjusting the tension of the incision through the locking part, it can effectively promote wound healing, reduce the occurrence of postoperative incision cracking, reduce mechanical tension of the incision, and avoid excessive keloid development (Chen et al., 2020). In addition to tension therapy, there are many other clinical treatments, including drug therapy (Yang et al., 2021), radiotherapy (Hsieh et al., 2021), cryotherapy (Lee et al., 2020), and stem cell therapy (Bojanic et al., 2021). Although there are many treatment options for keloids, any single treatment can lead to a higher recurrence rate, and multimodal combination therapy is a necessary and effective method at present. For example, after surgical resection, combined therapy with Z-plasty and electron beam irradiation to reduce wound tension can effectively control keloid locally (Wang et al., 2020).

## Conclusion

Keloids are a fibroproliferative disease caused by excessive growth of fibroblasts and excessive secretion of collagen. Many factors, including changes in cell function, genetic factors, and inflammation factors, play an important role in the occurrence and development of keloids. Biomechanical factors are a basic but relatively unexplored area for understanding keloids. Both macroscopic physical mechanical factors and microscopic matrix mechanical factors at a cellular or molecular level have been proved to have an important impact on the progression of keloid fibrosis and treatment research. Further study on the role of biomechanical factors in the keloid development and modification of mechanical forces or mechanical transduction signals will provide a direction for the development of new therapeutic strategies.

## Abbreviations

BMP, bone morphogenetic protein; CAV1, Caveolin-1; ECM, excessive extracellular matrix; EMT, epithelial-mesenchymal transition; EGF, epidermal growth factor; ET-1, endothelin-1; FGF-β, fibroblast growth factor β; GDF, growth differentiation factor; HATs, histone acetyl transferases; HCC, hepatocellular carcinoma; HDACs, histone deacetylases; IGF-I, insulin-like growth factor I; IL, interleukin; LOX, lysyl oxidase; MMP, matrix metalloproteinases; MSCs, mesenchymal stem cells; PDGF, platelet-derived growth factor; TAZ, transcroptional coactivator with a PDZ-binding domain; TGF-β, transforming growth factor -β; TIMPs, metalloproteinases; TMEM88, transmembrane protein 88; YAP, Yes-associated protein.
